# Exploring Ebola virus-associated gene expression through comparative analysis

**DOI:** 10.3389/fgene.2026.1793277

**Published:** 2026-04-17

**Authors:** Mostafa Rezapour, Sean V. Murphy, David A. Ornelles, Thomas D. Shupe, Stephen J. Walker, Alan R. Jacobson, Metin Nafi Gurcan, Patrick M. McNutt, Anthony Atala

**Affiliations:** 1 Wake Forest Institute for Regenerative Medicine, Wake Forest University School of Medicine, Winston-Salem, NC, United States; 2 Department of Microbiology Immunology, Wake Forest University School of Medicine, Winston-Salem, NC, United States; 3 Center for Artificial Intelligence Research, Wake Forest University School of Medicine, Winston-Salem, NC, United States

**Keywords:** differential gene expression analysis, Ebola virus, gene expression signature, host transcriptomic profiling, RNA sequencing

## Abstract

**Introduction:**

Ebola virus (EBOV) infection triggers intense host transcriptional responses that overlap extensively with those induced by other viral and bacterial pathogens. This overlap complicates the identification of EBOV-specific gene expression signatures and limits diagnostic specificity. Defining transcriptional markers that distinguish EBOV from other infections is essential for improving molecular diagnostics and advancing understanding of EBOV-specific host responses.

**Methods:**

We developed a multi-step filtering framework using blood-derived RNA-Seq data from nonhuman primates and human cohorts organized into independent training and test sets. In the training cohort, differential expression analysis was performed using an edgeR-based GLMQL-MAS approach to identify EBOV-associated genes. Candidates were filtered against non-EBOV comparator datasets, including mpox virus, influenza, bacterial pneumonia, acute HIV-1 infection, and multiple SARS-CoV-2 variants, to remove broadly shared host-response genes. Genes included in the NanoString nCounter® Host Response Panel were additionally excluded. The resulting EBOV-specific signature was evaluated in independent EBOV and non-EBOV test cohorts using principal component analysis and logistic regression. Functional enrichment was assessed using KEGG pathways.

**Results:**

Initial analysis identified numerous interferon-stimulated genes that were similarly upregulated across infections. After cross-infection filtering and NanoString exclusion, 281 EBOV-specific genes were identified. Optimization within the training cohort yielded a top-50 gene set that clearly separated EBOV from Non-EBOV samples. In the independent test cohort, classification performance improved substantially, with the F1 score increasing from 37.5% when all genes were used to 95.0% after applying the top-50 gene set. Enrichment analysis of the top-50 EBOV-specific genes revealed significant association with vascular, coagulation, secretory, and metabolic pathways. *ADAMTS1* showed consistent upregulation in EBOV while remaining downregulated or inactive in comparator infections.

**Discussion:**

Structured cross-pathogen filtering enables identification of EBOV-specific transcriptional features beyond shared antiviral responses. The validated gene signature generalizes across independent cohorts and highlights biologically distinct pathways, which supports its potential utility for host-based diagnostic development.

## 1 Introduction

Ebola virus (EBOV), a highly pathogenic member of the *Filoviridae* family, causes severe hemorrhagic fever in humans and non-human primates, with mortality rates reaching up to 90% in some outbreaks (Feldmann and Geisbert, 2011). Although supportive care and vaccine development have advanced, the mechanisms underlying host responses to EBOV infection remain only partially understood (Mulangu et al., 2019; Mehedi et al., 2011). Recent high-throughput transcriptomic studies have enabled comprehensive profiling of host gene expression during infection (Speranza et al., 2018; Cross et al., 2018).

However, a key challenge in EBOV research is identifying transcriptional changes that are truly specific to EBOV. While some studies have highlighted individual genes or gene families, particularly interferon-stimulated genes (ISGs) such as *ISG15* and *OASL* (Speranza et al., 2018), these markers are often upregulated across a range of viral infections (Perng and Lenschow, 2018; Melchjorsen et al., 2009), limiting their diagnostic specificity. Accurately defining EBOV-specific gene expression patterns is essential for improving diagnostic precision, characterizing virus-specific host responses, and distinguishing EBOV from other infectious diseases with overlapping immune signatures. Such gene sets could also guide the development of targeted therapeutics and early detection strategies for Ebola virus disease.

In this study, we aimed to define a set of EBOV-specific genes by systematically filtering out genes commonly regulated across other infections. We analyzed blood-derived RNA-Seq data from both nonhuman primates and human patients using a structured training–test framework. The training cohort consisted of an EBOV dataset used to identify candidate genes associated with EBOV infection, together with multiple non-EBOV comparator datasets, including Mpox virus (MPXV), influenza, bacterial pneumonia, acute HIV-1 infection, and several SARS-CoV-2 variants, which were incorporated to remove genes reflecting broadly shared antiviral and inflammatory host responses. To further enhance specificity during training, we excluded any genes included in the NanoString nCounter® Host Response Panel, which targets 773 immune-related genes commonly activated across diverse infections, such as those involved in interferon signaling and cytokine responses. Although these genes are effective in distinguishing infected from non-infected samples, their widespread regulation across pathogens limits their ability to differentiate EBOV from other infectious diseases.

The independent test cohort, structured similarly to the training cohort, included both EBOV and non-EBOV datasets that were entirely independent of those used during training. This cohort was used to evaluate whether the gene signature identified during training could accurately distinguish EBOV infection from non-EBOV infections in external datasets, thereby assessing its reproducibility and specificity across heterogeneous viral and bacterial host responses.

Together, this structured cross-pathogen filtering and validation strategy was designed to move beyond conventional differential expression analysis, which often captures generalized antiviral programs. Instead, our goal was to identify transcriptional features uniquely associated with EBOV infection through direct comparison with the targeted non-EBOV viral and bacterial datasets included in this study. By integrating statistical selection, cross-infection exclusion, NanoString panel filtering, independent validation, and downstream classification analysis, we aimed to derive a biologically meaningful and diagnostically specific EBOV-associated gene signature. The proposed framework is extensible, and additional pathogens can be incorporated as suitable high-quality datasets become available, which allows iterative refinement and strengthening of EBOV-specific transcriptional definitions over time.

## 2 Materials and methods

### 2.1 Data

To identify Ebola-specific host transcriptional signatures, we assembled a collection of blood-based RNA-Seq datasets derived from both EBOV infection and a wide range of non-Ebola viral and bacterial (non-EBOV) diseases. Ebola datasets were used to define candidate transcriptional responses associated with EBOV infection, whereas non-EBOV cohorts were incorporated to remove genes that reflect shared antiviral or inflammatory processes. Comparator infections were selected to represent distinct patterns of systemic host response. MPXV infection was included as a related zoonotic viral disease studied in nonhuman primates, which enabled assessment of transcriptional overlap across systemic viral infections. Influenza and multiple SARS-CoV-2 cohorts were incorporated because respiratory viral infections induce strong interferon-driven blood transcriptional signatures, which allowed exclusion of genes associated with generalized antiviral activation rather than EBOV-specific biology. Bacterial pneumonia datasets were included to control for transcriptional changes driven by non-viral inflammation. Acute HIV-1 infection was also incorporated because preliminary pathway analysis of candidate EBOV-associated genes revealed enrichment in HIV-related immune pathways, which made it possible to remove genes reflecting broader antiviral signaling instead of EBOV-specific responses.

All transcriptomic data were generated from *in vivo* models using peripheral whole blood or PBMC samples and sequenced using RNA-seq protocols. Although whole blood contains granulocyte populations that are largely absent from PBMC preparations, both sample types capture circulating immune transcriptional programs dominated by interferon signaling, inflammatory activation, and antiviral responses. To minimize biases arising from differences in sample source or study design, gene selection and differential expression analyses were performed independently within each dataset rather than on pooled expression matrices. Cross-cohort integration was applied only when visualization of global sample distributions or direct comparisons across cohorts were required, at which stage batch-effect correction was implemented to reduce technical variation while preserving biological signal.

All datasets were separated into training and held-out test cohorts to ensure strict independence between model development and validation.

#### 2.1.1 Training datasets

EBOV training dataset: Speranza et al. (2018) conducted a controlled infection study using twelve healthy cynomolgus macaques (*Macaca fascicularis*), challenged intranasally with 100 PFU of the EBOV/Makona strain using either a pipette or a mucosal atomization device (MAD). The animals were monitored over a 41-day period, and blood samples were collected longitudinally at multiple time points post-infection. Whole blood was used for transcriptomic profiling. Total RNA was isolated from samples preserved in TRIzol LS and processed using the PureLink RNA Mini Kit. Sequencing was performed on the Illumina HiSeq 2,500 using paired-end 2 × 100 bp reads. Viral RNA levels were also quantified by Speranza et al. (2018) using RT-qPCR from plasma samples mixed with TRIzol LS and processed with either the QIAamp Viral RNA Mini Kit or the EZ1 Virus Mini Kit. An EBOV-specific RT-qPCR assay with a synthetic RNA standard curve was used to measure genome equivalents per milliliter (GSE103825 (Speranza et al., 2018)).

For the current study, we used RNA-Seq data from all 12 animals on day 0 post-infection (DPI 0) as the baseline group (control) and selected 12 samples that tested positive by RT-qPCR at later time points as the EBOV-positive group for statistical analysis.

Non-EBOV training datasets: To remove genes associated with shared host responses across infections, multiple non-EBOV RNA-Seq cohorts were incorporated during model development. Whole-blood RNA-Seq samples from Indian-origin rhesus macaques (*Macaca mulatta*) infected with the 2022 hMPXV/USA/MA001/2022 strain (Clade 2b, Lineage B.1) were included (GSE234118 (Rashwan et al., 2025)). Animals were stratified at the individual level to prevent information leakage, and samples derived from animals T461M, T462M, T448F, T441F, T409F, T445F, T437F, T443F, and T465M were assigned to the training datasets.

Human peripheral blood RNA-Seq datasets from PCR-confirmed influenza and bacterial pneumonia cases were included (GSE161731 (McClain et al., 2021)). Additional SARS-CoV-2 cohorts were incorporated to account for diverse coronavirus-associated immune responses, including whole-blood RNA-Seq samples from severe COVID-19 ICU patients and COVID-negative controls in France (GSE171110 (Lévy et al., 2021)), PBMC samples from COVID-positive (Beta variant) and COVID-negative individuals in Italy (GSE189039 (Knabl et al., 2022)), and whole-blood RNA-Seq data from Omicron-infected and COVID-negative individuals in Austria (GSE201530 (Lee et al., 2022)).

Whole-blood RNA-Seq data from individuals with acute HIV-1 infection and uninfected controls were also included (GSE199911 (Parker et al., 2023)). This cohort consisted of subjects sampled approximately 1 month after presentation alongside contemporaneous healthy individuals. Control and HIV-positive samples were randomly divided in a stratified manner, with 23 control samples and 14 HIV-infected samples incorporated into the training datasets.

#### 2.1.2 Held-out test datasets

EBOV test dataset: Cross et al. (2018) investigated EBOV infection in healthy rhesus macaques (*Macaca mulatta*), which were challenged intramuscularly with 1000 PFU of the EBOV Makona C05 isolate. Blood samples were collected at designated time points (days 0, 5, and 7 post-infection), as well as at necropsy from terminal animals. RNA was extracted from whole blood samples, and quality was assessed using the Agilent Bioanalyzer 2,100. Ribosomal RNA was depleted using the Illumina TruSeq Stranded RNA RZ Gold Kit, and libraries were constructed using 500 ng of input RNA. Sequencing was carried out on the Illumina NextSeq 500 platform using paired-end 50 bp reads, with adapter trimming and demultiplexing performed via the Illumina BaseSpace platform (GSE115785 (Cross et al., 2018)). Library concentrations were quantified using HeLa S3 RNA-Seq standards and qPCR.

For simplicity, the RNA-Seq data collected on days 0, 5, and 7 post-infection, as well as at necropsy from terminal animals, are hereafter referred to as DPI 0 (baseline), DPI 5, DPI 7, and DPI NEC, where “DPI NEC” denotes samples collected at necropsy from terminal animals. Note that in the current study, the test dataset was used solely to validate the results derived from the Ebola training dataset (Speranza et al., 2018). However, when statistical analysis was required, animals at DPI 0 were used as the baseline.

Non-EBOV test datasets: Independent non-EBOV cohorts were used to evaluate whether candidate EBOV-associated transcriptional signatures remained specific across diverse host responses. Peripheral whole-blood RNA-Seq datasets from multiple seasonal influenza cohorts were included (GSE158592, GSE155635, GSE196350, and GSE213168 (Schughart et al., 2024)), representing independent transcriptome batches collected between 2018 and 2022. These cohorts contained PCR-confirmed influenza cases sampled at hospital admission or ICU enrollment alongside non-infected healthy individuals.

Whole-blood RNA-Seq data from PCR-confirmed SARS-CoV-2-positive patients with mild to moderate disease and healthy controls were also incorporated (GSE161731 (McClain et al., 2021)). Participants were sampled longitudinally and categorized into early, middle, and late stages following symptom onset, with all infected samples treated as SARS-CoV-2-positive for the present analysis.

A multinational host-response cohort comprising adjudicated bacterial and influenza infections was included to assess transcriptional overlap with non-EBOV inflammatory responses (GSE211567 (Ko et al., 2023)). In addition, a clinical whole-blood transcriptomic dataset comparing SARS-CoV-2 infection, influenza infection, and healthy controls was incorporated (PMC8202013 (Bibert et al., 2021)).

To maintain strict separation between model development and evaluation, held-out subsets from previously introduced cohorts were reserved exclusively for testing. These included MPXV samples derived from animals T456M, T464M, T411F, T439F, T447F, T442F, T433F, T438F, and T444F from GSE234118 (Rashwan et al., 2025), as well as an independent subset from the acute HIV-1 cohort (GSE199911 (Parker et al., 2023)) consisting of 23 control samples and 14 HIV-infected samples from subjects not included in the training datasets.

### 2.2 Methodology

#### 2.2.1 Training-stage pipeline

**FIGURE 1:**
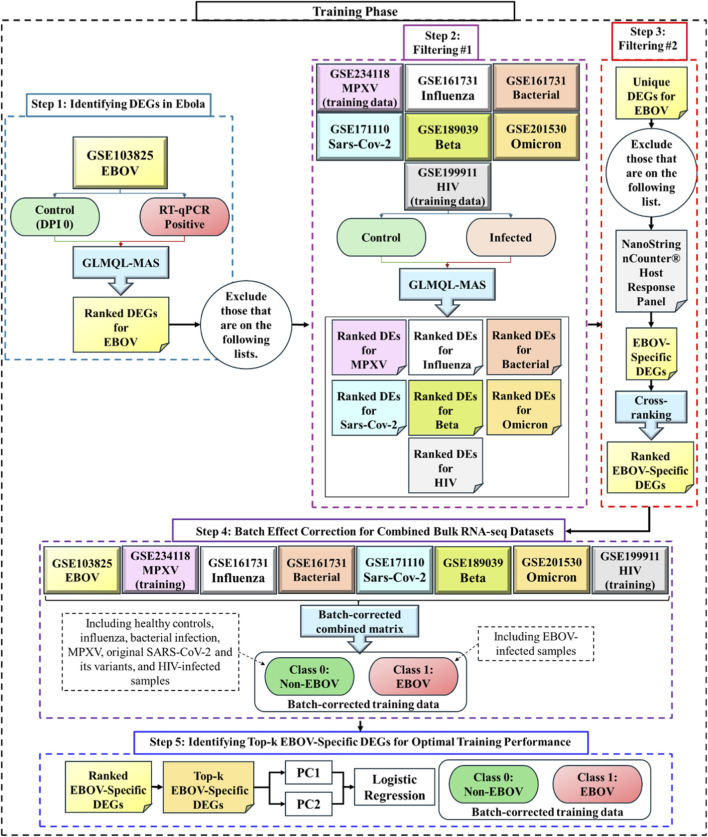
Overview of the five-step training-stage pipeline used to define and evaluate EBOV-specific transcriptional signatures. Step 1 identifies differentially expressed genes in the EBOV training dataset using the GLMQL-MAS framework, which integrates generalized linear modeling, quasi-likelihood F-tests, and Magnitude–Altitude Scoring. Step 2 applies the same approach to non-EBOV filtering datasets to remove genes associated with shared host responses across infections. Step 3 excludes genes present in the NanoString nCounter® Host Response Panel and prioritizes remaining candidates using a cross-ranking strategy based on raw p -value non-significance in non-EBOV cohorts followed by EBOV-derived MAS re-ranking, yielding a ranked list of EBOV-specific genes. Step 4 concatenates datasets after gene selection, applies TMM normalization and limma-based batch-effect correction to enable cross-cohort visualization and comparison, and constructs a combined training matrix labeled as EBOV or Non-EBOV. Step 5 evaluates discriminatory performance using PCA-derived features and logistic regression to determine the optimal number of EBOV-specific genes for classification.

Our training-stage pipeline consists of five steps (see Figure 1 for an overview). In step 1, we used the EBOV-training dataset, and employed Generalized Linear Models with Quasi-Likelihood F-tests and Magnitude-Altitude Score (GLMQL-MAS) technique (Rezapour et al., 2025a; Rezapour et al., 2024a; Rezapour et al., 2025b), which is an edgeR-based (Chen et al., 2025) method and combines Generalized Linear Models (GLMs) (Nelder and Wedderburn, 1972) with Quasi-Likelihood F-tests (Wedderburn, 1974) and Magnitude-Altitude Scoring (MAS) (Rezapour et al., 2024b; Rezapour et al., 2024c; Rezapour et al., 2025c) to identify differentially expressed genes. GLMs are well-suited for RNA-Seq count data due to their ability to model overdispersion (Law et al., 2014).

Experimental groups were defined using DPI 0 samples as controls and RT-qPCR positive samples as EBOV-infected in the training dataset. A design matrix was constructed to model all conditions, and a GLM was applied to the trimmed mean of M values (TMM) (Robinson and Oshlack, 2010) normalized data with dispersion estimates to account for variability.

Quasi-Likelihood F-tests compared a full model (control + infected) to a reduced model (control only), yielding log fold change (LogFC) values, p-values and False Discovery Rate (FDR). We applied the Benjamini-Hochberg (BH) method (Benjamini et al., 2009; Benjamini and Hochberg, 1995) with α=0.05 and retained genes with $\left|LogFC\right|\;>\;1$ ( LogFC > 1 or LogFC <−1 ). MAS integrates both $\left|LogFC\right|\;>\;1$ and adjusted p-values:

$${\text{MAS}}_{l}={\left|\log_{2}\left({FC}_{1}\right)\right|}^{M}{\left|\log_{10}\left(P_{1}^{BH}\right)\right|}^{A}$$ (1)

where $p_{l}^{BH}$ denotes BH adjusted p-values (FDR) for l =1,2,…,s , where s is the number of rejected null hypotheses based on BH adjusted p-value ( $\left.p_{l}^{BH}<\alpha =0.05\right)$ . Parameters M and A are set to 1 in this study. MAS was used as a ranking metric to prioritize differentially expressed genes within each contrast, which integrates effect size and statistical significance into a single score.

In step 2, we again applied the GLMQL-MAS approach to identify and rank BH-significant genes with $\left|LogFC\right|\;>\;1$ for all filtering datasets. For MPXV, DPI 0 samples served as controls, while DPIs 3, 7, 10 and 14 samples were each contrasted against DPI 0 group separately, resulting in four distinct gene lists. This same procedure was repeated for influenza, bacterial pneumonia, SARS-CoV-2 (original strain, Beta, and Omicron variants) and HIV, treating healthy individuals within each dataset as controls and infected individuals as treated groups. After compiling the BH-significant genes with $\left|LogFC\right|\;>\;1$ from all non-EBOV datasets, we removed them from the EBOV gene list identified in step 1.

In step 3, we further refined the list by excluding any genes included in the NanoString nCounter® Host Response Panel, yielding a set of EBOV-specific differentially expressed genes (EBOV-specific DEGs). This panel targets a broad spectrum of 773 immune-related genes commonly dysregulated across various infections. While effective in distinguishing infection status, these genes are involved in conserved immune responses triggered by many pathogens, and their removal improves the specificity of the EBOV signature.

The resulting EBOV-specific DEGs included both upregulated and downregulated genes, which were subsequently prioritized using a cross-ranking strategy inspired by the Cross-MAS ranking system (see Algorithm 2 in Rezapour et al. (2024d)). Genes were first ranked based on raw p -values obtained from non-EBOV filtering datasets, without multiple-testing correction and independent of log fold change thresholds. Candidates were prioritized when they consistently exhibited raw p -values >0.05 across comparator infections. This criterion ensured that selected genes remained statistically non-significant in other disease contexts under nominal testing conditions. For each regulation direction, the top-ranked candidates were then re-ranked using the EBOV-derived MAS (see Equation 1), which integrates absolute log fold change and adjusted statistical significance. This two-stage ranking procedure produced a prioritized list of ranked EBOV-specific genes, in which top-ranked candidates exhibited strong EBOV-associated differential expression while remaining minimally responsive in non-EBOV conditions, which enhanced the specificity of the final candidate gene set.

Up to this stage, all gene selection procedures were performed independently within each dataset to avoid introducing cross-study bias. In step 4, training datasets were concatenated only after identification and ranking of EBOV-specific genes to enable cross-cohort visualization and downstream classification analyses. Raw count matrices from all training cohorts were first restricted to genes shared across datasets and then merged into a unified expression matrix. Library size normalization was performed using the TMM method implemented in edgeR (v4.4.2), followed by transformation to log-counts per million (logCPM).

Because samples originated from multiple independent studies with different experimental conditions and sequencing platforms, batch-effect correction was applied using the *removeBatchEffect* function (Ritchie et al., 2015) in the limma package (v3.62.2). This approach was selected because it provides a robust modeling strategy for reducing dataset-specific technical variation while preserving biologically meaningful signal, which makes it well suited for joint visualization and comparison after gene selection.

The resulting batch-corrected expression matrix was used to construct a combined training dataset. Within this normalized matrix, all RT-qPCR–positive samples were labeled as EBOV (class 1), whereas all remaining samples, including non-EBOV infections and healthy controls, were labeled as Non-EBOV (class 0).

In step 5, we assessed the discriminatory performance of the EBOV-specific gene set. Principal component analysis (PCA) was performed using the top- k ranked EBOV-specific genes, and the first two principal components (PC1 and PC2) were used as predictors in a logistic regression model to evaluate how effectively the selected genes distinguished EBOV samples from Non-EBOV samples.

The value of *k* was determined exclusively within the training dataset by evaluating classification performance as a function of *k* using PC1 and PC2, and selecting the smallest *k* that achieved optimal separation, thereby avoiding information leakage. This strategy was designed to minimize overfitting (Ying, 2019) and enhance the generalizability of the results. By reducing the feature space to two orthogonal components, PC1 and PC2, that capture the majority of variance, we constrained model complexity while preserving key discriminatory information. Logistic regression, a simple and interpretable linear model with inherently low variance, was selected to avoid the overfitting risks associated with more complex models (Ying, 2019).

#### 2.2.2 Test-stage evaluation

**FIGURE 2:**
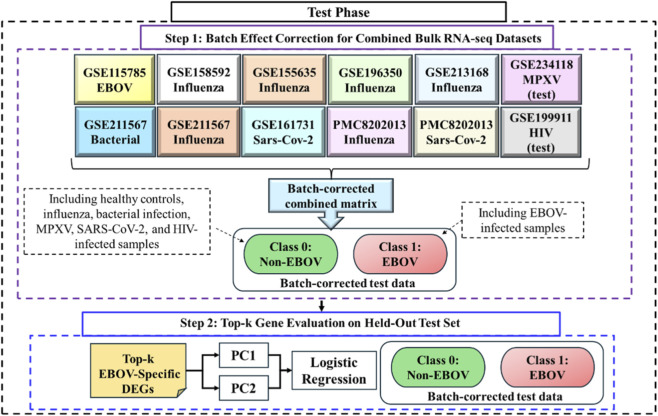
Overview of the two-step independent test-stage evaluation pipeline. In step 1, all test cohorts were concatenated following the same normalization and batch-correction procedure described for the training stage, including restriction to shared genes, TMM normalization, logCPM transformation, and limma-based batch-effect correction to enable joint analysis across heterogeneous datasets. NHP samples collected at DPI 5, DPI 7, and necropsy (DPI NEC) were labeled as EBOV (class 1), whereas baseline samples and all non-EBOV infection cohorts were labeled as Non-EBOV (class 0). In step 2, the top- k EBOV-specific genes identified during training were applied without re-selection, and principal component analysis was performed. The first two principal components (PC1 and PC2) served as predictors in a logistic regression model to evaluate the ability of the training-derived gene signature to distinguish EBOV from Non-EBOV samples in independent test datasets.

The independent test-stage evaluation consisted of two steps (see Figure 2 for an overview). In step 1, all test datasets were concatenated following the same normalization and batch-correction procedure described in step 4 of the training stage. In step 2, the top- k EBOV-specific genes identified during the training stage were applied directly to the batch-corrected test matrix without re-selection or re-ranking. PCA was performed using these genes, and the first two principal components (PC1 and PC2) served as predictors in a logistic regression model to evaluate classification performance on independent data. In the test cohort, NHP samples collected at DPI 5, DPI 7, and DPI NEC were labeled as EBOV (class 1), whereas all remaining samples, including non-EBOV infections and healthy controls, were labeled as Non-EBOV (class 0).

#### 2.2.3 Functional enrichment analysis

To investigate the biological relevance of the top-*k* EBOV-specific genes, functional enrichment analysis was performed using the Enrichr platform (Kuleshov et al., 2016). The top-*k* EBOV-specific gene set was submitted to Enrichr, and enrichment was evaluated against the KEGG 2021 Human pathways and Gene Ontology (GO) Biological Process 2021 libraries. For visualization, enriched pathways and biological processes were ranked according to statistical significance, and bar plots were generated using the $-\log_{10}\left(p‐\text{value}\right)$ metric to highlight the most strongly associated functional categories.

## 3 Results

### 3.1 Training-stage identification of EBOV-specific transcriptional signatures

**FIGURE 3:**
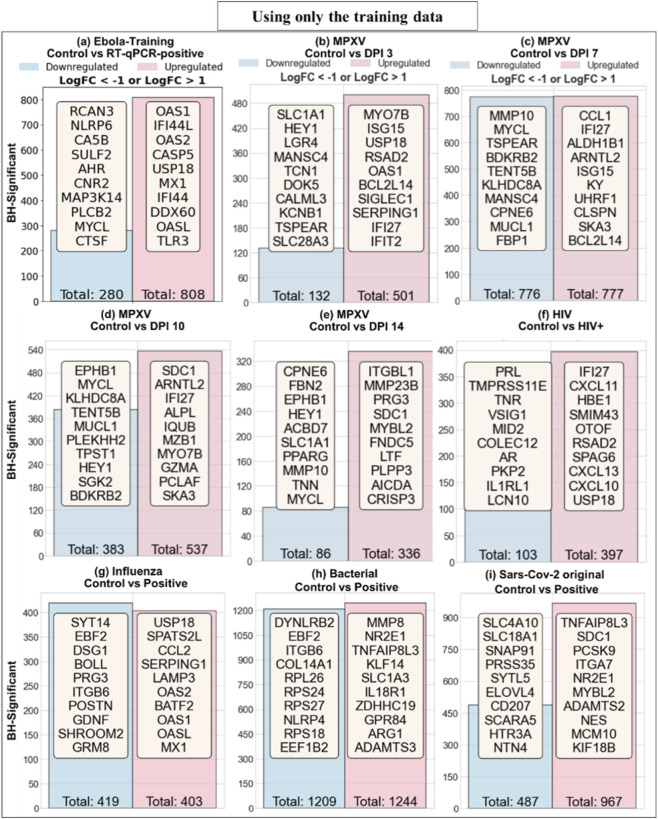
Number of BH-significant genes with |LogFC| > 1 identified using GLMQL-MAS across datasets. Panel **(a)** shows results for the Ebola training dataset. Panels **(b–e)** present results for MPXV at DPIs 3, 7, 10, and 14. Panels **(f–i)** show gene counts for HIV, influenza, bacterial pneumonia, and SARS-CoV-2 (original), respectively.

Figure 3 shows the total number of BH-significant genes with $\left|LogFC\right|\;>\;1$ identified using the GLMQL-MAS approach across the training dataset and all filtering datasets. For each contrast, differentially expressed genes were subsequently ranked according to their MAS. This figure shows that a wide range of immune-related genes are significantly upregulated or downregulated not only during EBOV infection but also across other infections. This widespread overlap underscores the challenge of identifying genes uniquely associated with EBOV and highlights the need for a stringent filtering strategy to remove commonly regulated genes. Note that, among the MPXV time points, DPI 7 showed the highest number of significant genes, which suggests a peak in host transcriptional response at this stage of infection.

**FIGURE 4:**
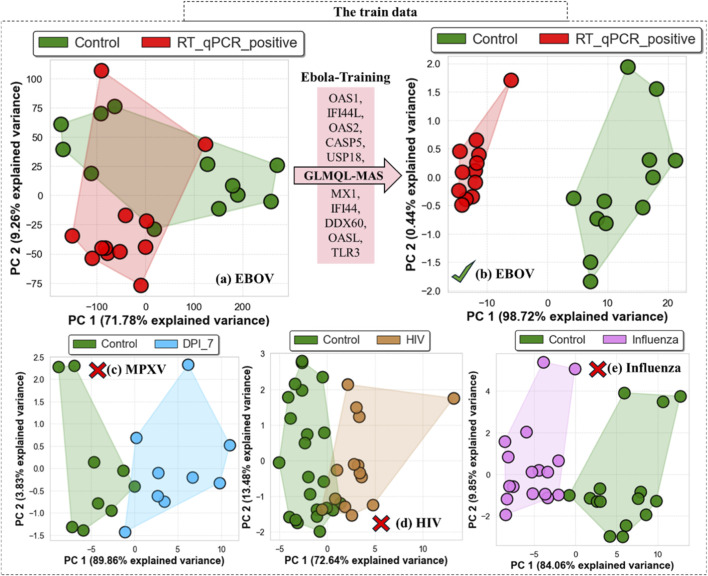
Principal component analysis (PCA) of gene expression profiles using the top 10 GLMQL-MAS selected genes from the Ebola training dataset. Panel **(a)** shows the distribution of control and RT-qPCR positive samples before applying GLMQL-MAS, based on all genes. Panel **(b)** shows the separation between the same groups after applying GLMQL-MAS and using only the top 10 selected genes: OAS1, IFI44L, OAS2, CASP5, USP18, MX1, IFI44, DDX60, OASL, and TLR3. Panels **(c–e)** display the distribution of control versus MPXV (DPI 7), HIV and influenza-positive samples, respectively, using the same 10 genes.

Figure 4 shows that the top 10 GLMQL-MAS selected genes from the Ebola training dataset, *OAS1*, *IFI44L*, *OAS2*, *CASP5*, *USP18*, *MX1*, *IFI44*, *DDX60*, *OASL*, and *TLR3*, can differentiate not only between EBOV-infected and non-infected samples (Figure 4b), but also observable separation between infected and non-infected samples in MPXV (Figure 4c), HIV (Figure 4d) and influenza (Figure 4e) datasets. These genes are highly effective for identifying infection status; however, they lack EBOV specificity, as all of them are significantly upregulated in response to both EBOV and influenza, making them unsuitable for distinguishing between these two infections.

**FIGURE 5:**
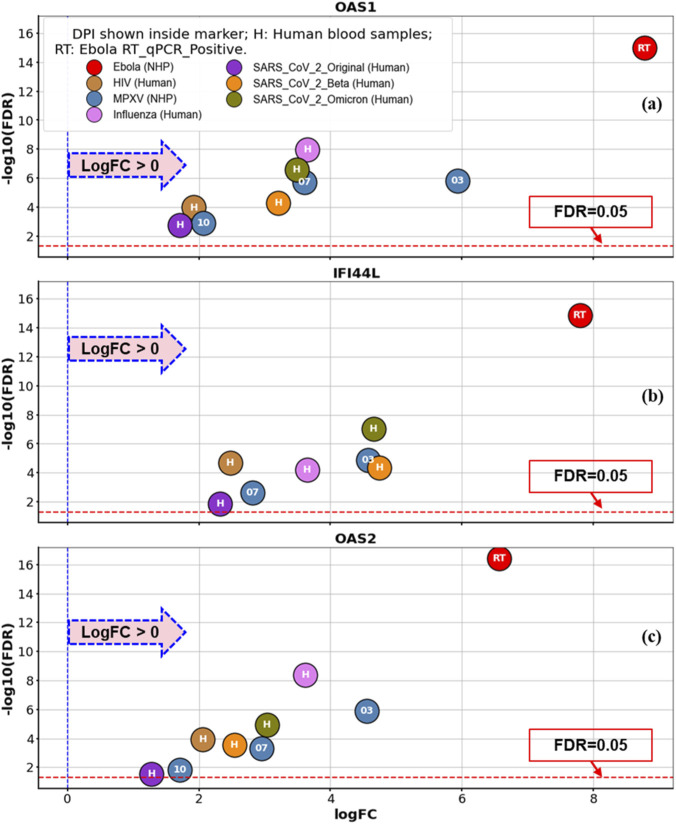
Specific volcano plots. This figure shows the differential expression of **(a)** OAS1, **(b)** IFI44L and **(c)** OAS2 across EBOV and other infection datasets. Each point represents a disease condition, with the x-axis indicating the log fold change (LogFC) and the y-axis showing -log10(FDR).

Figure 5 presents specific volcano plots (LogFC vs. -Log10(FDR)) for the top three GLMQL-MAS selected genes from the Ebola training dataset, *OAS1*, *IFI44L*, and *OAS2*. For each gene, the figure illustrates differential expression results from comparisons between infected and control groups across multiple datasets, including EBOV (RT-qPCR positive vs. control), MPXV, HIV, influenza, and three SARS-CoV-2 variants. In all cases, these genes are significantly upregulated in infected samples. These results again highlight that while *OAS1*, *IFI44L*, and *OAS2* are strong indicators of infection, their consistent upregulation across diverse pathogens limits their utility as EBOV-specific markers.

**FIGURE 6:**
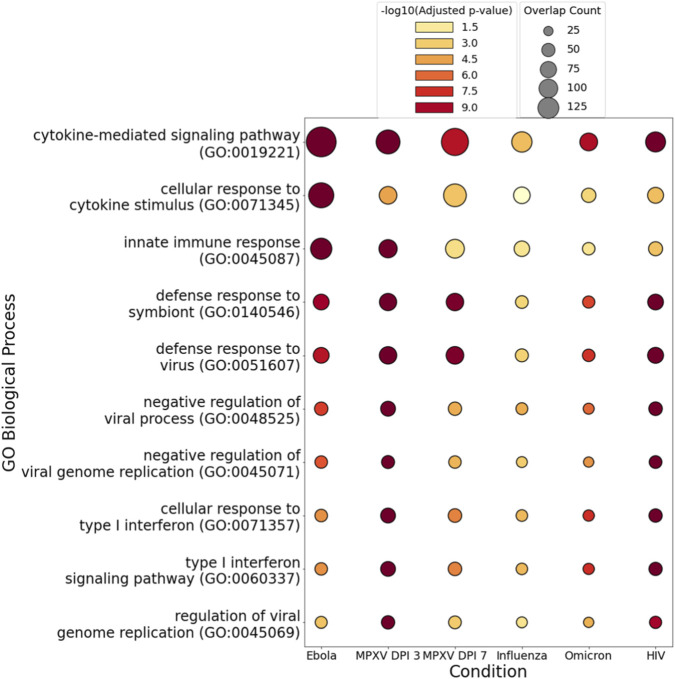
Bubble plot shows the top GO Biological Process terms commonly enriched across EBOV, MPXV (DPI 3 and DPI 7), influenza, SARS-CoV-2 Omicron, and HIV infections. Each bubble represents a significantly enriched GO term, where the color indicates statistical significance (-log10 of the adjusted p-value) and the size reflects the number of overlapping genes contributing to that term within each condition. Warmer colors (e.g., red) represent more significant enrichment, while larger bubbles indicate greater gene overlap.

Figure 6 highlights the top GO Biological Process terms that are significantly enriched across EBOV, MPXV (DPI 3 and DPI 7), influenza, SARS-CoV-2 Omicron, and HIV infections. Notably, key immune-related biological processes such as cytokine-mediated signaling, innate immune response, and defense response to virus are consistently activated across these conditions. These shared GO terms involve a recurrent set of host response genes, including *MX1*, *OAS1*, *OAS2*, *OASL*, *IRF7*, *RSAD2*, *IFIT5*, and *STAT2*, which appear across multiple processes and infections. The broad and repeated activation of these genes underscores a common antiviral and inflammatory response rather than a virus-specific signature.

Results presented in Figures 4–6 collectively emphasize the necessity of implementing a filtering strategy to exclude genes that are broadly responsive to infection, and ensure that the final gene set reflects transcriptional changes specific to EBOV.

**FIGURE 7:**
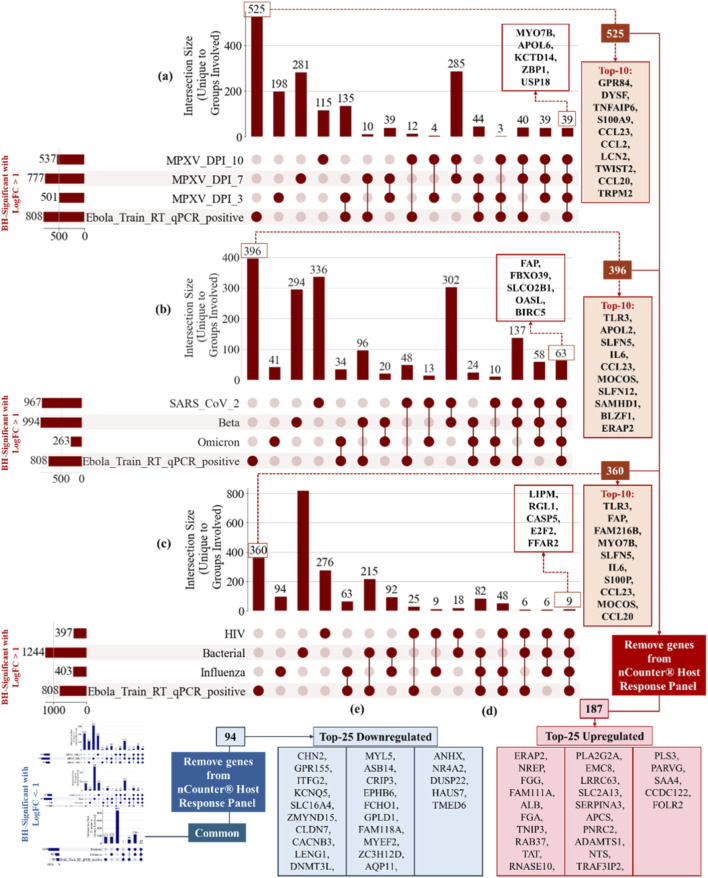
Filtering strategy to identify EBOV-specific genes. Panels **(a–c)** show UpSet plots and intersection bar graphs summarizing the overlap of upregulated BH-significant genes (LogFC >1) between EBOV and **(a)** MPXV at multiple DPIs, **(b)** SARS-CoV-2 variants (original, Beta, Omicron), and **(c)** influenza, bacterial and HIV infections. Vertical bars represent the number of significant genes that are unique to or shared across datasets, while horizontal bars indicate the total number of significant genes identified in each condition. Panels **(d)** and **(e)** show the top 25 upregulated and top 25 downregulated EBOV-specific genes, respectively, after applying both filtering steps.

Figure 7 displays the details of the filtering system. By applying the GLMQL-MAS method and the Cross-MAS (see Algorithm 2 in (Rezapour et al., 2024d)), Figures 7a–c illustrate the number of common and unique upregulated BH-significant genes with LogFC > 1 between EBOV and (a) MPXV at different DPIs, (b) various SARS-CoV-2 variants, and (c) influenza, bacterial and HIV infections. After identifying genes that were significant only for EBOV and not for any of the other infections, we further removed those included in the NanoString nCounter® Host Response Panel, resulting in 187 upregulated EBOV-specific genes. Figure 7d presents the top 25 of these genes after applying the Cross-ranking system. Applying the same procedure to BH-significant genes with LogFC <−1 yielded 94 downregulated genes that passed both filtering steps, with the top 25 shown in Figure 7e. For downstream classification tasks, the number of top genes (*k*) was optimized during the training phase, and the combination of the top 25 upregulated and top 25 downregulated genes (k = 50), as shown in Figures 7d,e, provided the optimal classification performance. These genes are hereafter collectively referred to as the *top-50 EBOV-specific gene* set used for classification.

**FIGURE 8:**
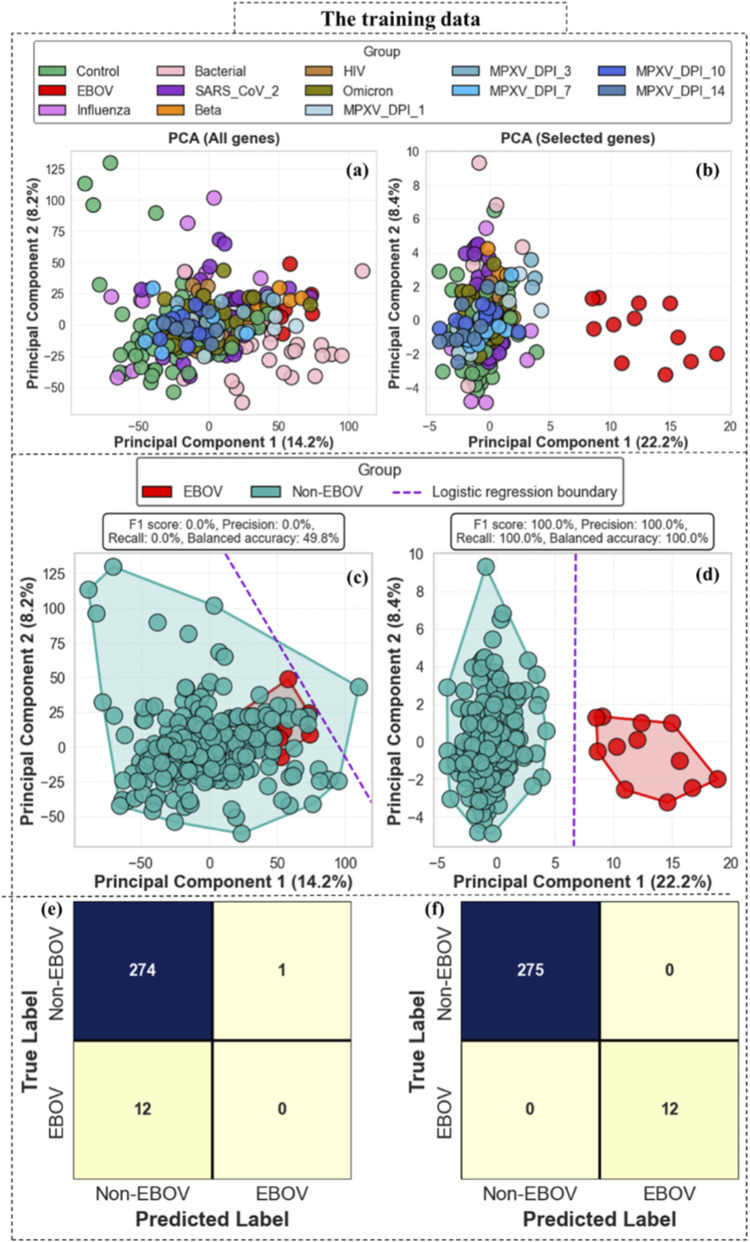
Effect of EBOV-specific gene selection on sample separation within the training cohort. **(a)** PCA was performed using all genes shows substantial overlap between EBOV and Non-EBOV samples. **(b)** PCA based on the top-50 EBOV-specific genes demonstrates improved separation along the first two principal components (PC1 and PC2). **(c,d)** Logistic regression decision boundaries derived from PC1 and PC2 illustrate limited discrimination when all genes are used **(c)**, compared with clearer separation achieved using the selected EBOV-specific genes **(d)**. **(e,f)** Corresponding confusion matrices highlight the improvement in classification performance after gene prioritization, where the model using the top-50 EBOV-specific genes shows complete separation of EBOV and Non-EBOV samples within the training dataset.

Figure 8 illustrates the effect of EBOV-specific gene selection within the training cohort. Panels (a) and (c) show principal component analysis and logistic regression results obtained using all genes, where EBOV and Non-EBOV samples display substantial overlap and limited separation along PC1 and PC2. In contrast, panels (b) and (d) present results derived from the top-50 EBOV-specific genes, where the first two principal components provide clear separation between EBOV and Non-EBOV samples. The corresponding confusion matrices in panels (e) and (f) further demonstrate the improvement achieved after gene prioritization, with the model based on all genes showing poor EBOV detection performance, whereas the model using the top-50 EBOV-specific genes achieves complete separation of EBOV and Non-EBOV samples within the training dataset. These findings indicate that prioritization of EBOV-specific genes substantially enhances separability while reducing the influence of shared host-response signals across infections.

### 3.2 Independent test-stage validation of EBOV-specific genes

**FIGURE 9:**
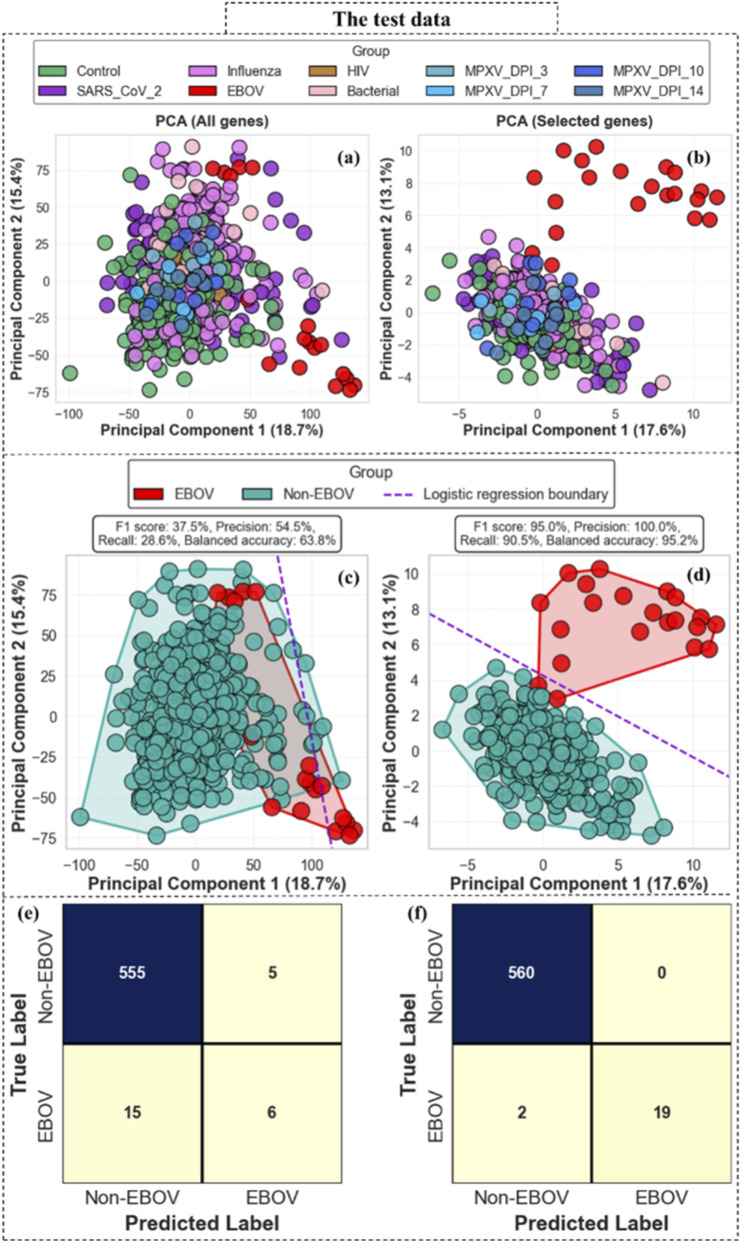
Independent test-stage PCA and classification performance using all genes versus the top-50 EBOV-specific genes. **(a,b)** PCA plots show sample distribution in the test cohort using all genes **(a)** and the selected EBOV-specific genes **(b)**. **(c,d)** Logistic regression boundaries derived from PC1 and PC2 illustrate improved separation after application of the EBOV-specific gene set. **(e,f)** Confusion matrices comparing classification performance before and after gene prioritization in the independent test datasets.

To evaluate whether the EBOV-specific gene signature generalizes beyond the training data, we applied the top-50 EBOV-specific genes to the independent test cohorts. Figure 9 compares sample separation and classification performance obtained using all genes versus the selected EBOV-specific gene set. PCA based on all genes shows substantial overlap between EBOV and Non-EBOV samples, which indicates limited discriminative structure in the full transcriptome space (Figure 9a). In contrast, PCA derived from the top-50 EBOV-specific genes demonstrates clearer separation between EBOV and Non-EBOV groups along PC1 and PC2 (Figure 9b). Logistic regression boundaries derived from these components further highlight this difference, where the model using all genes exhibits reduced sensitivity for EBOV detection (Figure 9c), whereas application of the EBOV-specific gene set results in markedly improved separation and classification performance in the independent test cohort (Figure 9d). Consistent with these observations, the F1 score increased from 37.5% when principal components (PC1 and PC2) were constructed using all genes and used as predictors in the logistic regression model, to 95.0% when PC1 and PC2 were derived from the top-50 EBOV-specific genes and used in the same classification framework. The corresponding confusion matrices (Figures 9e,f) further support this improvement, which indicates that the training-derived gene signature captures EBOV-associated transcriptional patterns that extend to external datasets.

**FIGURE 10:**
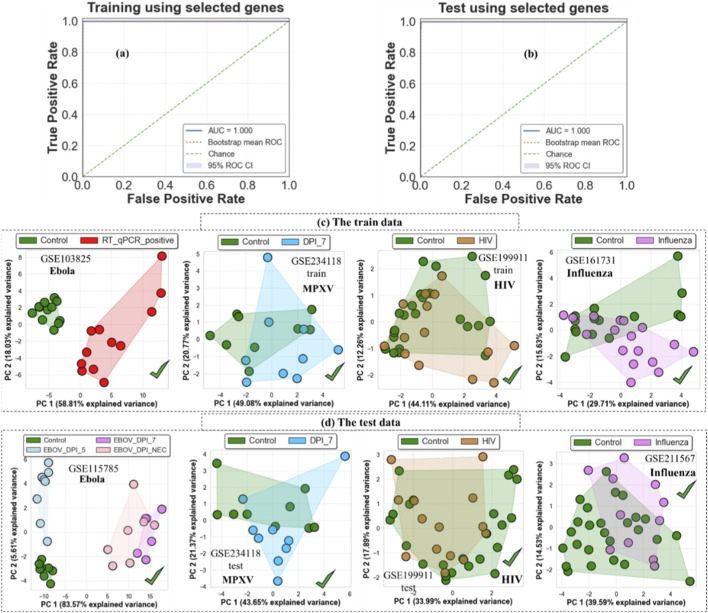
ROC–AUC performance and dataset-specific separation using the top-50 EBOV-specific genes. **(a,b)** ROC curves show EBOV versus Non-EBOV classification performance in the training **(a)** and independent test **(b)** cohorts. **(c)** PCA plots for individual training datasets demonstrate separation of EBOV-infected samples from controls, whereas non-EBOV infections show substantial overlap with healthy samples. **(d)** Independent test datasets display a similar pattern, which supports the EBOV-specific nature of the selected gene set.

Figure 10 further evaluates the performance of the top-50 EBOV-specific genes across both combined and individual datasets. Receiver operating characteristic (ROC) curves derived from the training and independent test cohorts demonstrate strong EBOV versus Non-EBOV discrimination when all pathogens are considered together, as reflected by the high AUC values shown in panels (a) and (b). To assess whether these genes capture EBOV-specific rather than general infection signals, PCA was examined separately for each dataset. Within the EBOV training cohort, the selected genes clearly separate EBOV-infected samples from controls (panel (c)); however, the same genes do not produce comparable separation between control and non-EBOV infections such as MPXV, HIV, or influenza when analyzed individually. A similar pattern is observed in the independent test datasets (panel (d)), where EBOV samples remain distinct from controls while other pathogens exhibit substantial overlap with healthy samples. Together, these results indicate that the selected genes capture transcriptional features associated with EBOV infection specifically, rather than reflecting a generic host-response signature.

**FIGURE 11:**
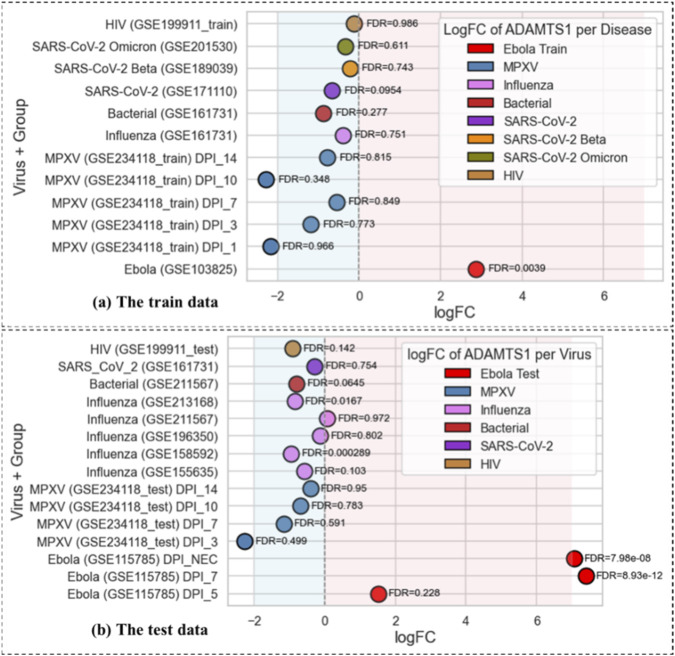
Disease-specific differential expression pattern of ADAMTS1 across training and test datasets. Log fold change (logFC) values for ADAMTS1 are shown across EBOV and non-EBOV infections in the training **(a)** and independent test **(b)** cohorts. ADAMTS1 is consistently upregulated in EBOV samples, whereas most non-EBOV infections display neutral or downregulated expression, which highlights its potential contribution to EBOV-specific transcriptional signatures.

To further illustrate the disease-specific behavior of the selected gene set, Figure 11 highlights the differential expression pattern of *ADAMTS1* across training and independent test datasets. In both cohorts, *ADAMTS1* shows strong upregulation in EBOV samples, whereas non-EBOV infections predominantly exhibit downregulated expression, with only a single dataset displaying expression levels close to baseline. Most comparator pathogens show non-significant adjusted p -values, which indicates minimal transcriptional activation relative to controls. When statistical significance is observed for non-EBOV infections, the direction of change corresponds to downregulation rather than upregulation. This consistent pattern across datasets suggests that *ADAMTS1* may contribute to EBOV-associated transcriptional responses; however, its biological role and mechanistic relevance require further investigation.

### 3.3 Functional enrichment analysis

**FIGURE 12:**
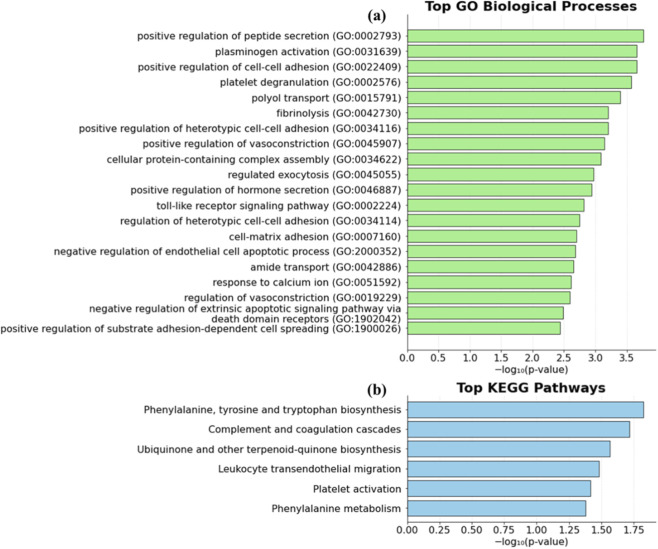
Functional enrichment analysis of the top-50 EBOV-specific genes. **(a)** Gene Ontology (GO) Biological Process enrichment results derived from the top-50 EBOV-specific gene set. Bars represent enriched biological processes ranked by statistical significance. **(b)** KEGG pathway enrichment analysis showing top pathways associated with the EBOV-specific gene signature.

To investigate the biological relevance of the prioritized EBOV-specific transcriptional signature, functional enrichment analysis was performed using the top-50 EBOV-specific genes identified during the training-stage pipeline. Enrichment results are summarized in Figure 12.

Gene Ontology (GO) Biological Process analysis identified a defined set of significantly enriched functional categories (Figure 12a). The most strongly enriched processes included positive regulation of peptide secretion and plasminogen activation. Multiple adhesion-related processes were also overrepresented, including positive regulation of cell-cell adhesion, regulation of heterotypic cell-cell adhesion, and cell-matrix adhesion. Enrichment was additionally observed for vascular and endothelial-associated processes, such as regulation of vasoconstriction and negative regulation of endothelial cell apoptotic process. Immune-related signaling was reflected by enrichment of the toll-like receptor signaling pathway. Terms such as response to calcium ion, regulated exocytosis, and cellular protein-containing complex assembly further indicate involvement of intracellular signaling and secretory mechanisms.

KEGG pathway enrichment analysis of the top-50 EBOV-specific genes (Figure 12b) revealed significant enrichment of pathways related to complement and coagulation cascades, platelet activation, and leukocyte transendothelial migration, which collectively point to coordinated immune and vascular activity. Metabolic pathways were also enriched, including phenylalanine, tyrosine and tryptophan biosynthesis, phenylalanine metabolism, and ubiquinone and other terpenoid-quinone biosynthesis. The concurrent enrichment of vascular, immune, and metabolic pathways suggests that the EBOV-specific transcriptional profile may reflect host responses that involve vascular, inflammatory, and metabolic processes.

Together, the GO and KEGG enrichment results suggest that the top-50 EBOV-specific genes may be associated with secretion, adhesion, vascular regulation, coagulation, and immune signaling, with additional links to selected metabolic pathways. The enrichment of processes such as plasminogen activation, complement and coagulation cascades, and platelet activation may indicate involvement of coordinated vascular and inflammatory mechanisms. Overall, these findings provide functional context for the selected genes and may reflect EBOV-associated host responses that extend beyond broadly shared antiviral programs.

## 4 Discussion

In this study, we developed and validated a systematic filtering strategy to identify EBOV-specific transcriptional signatures using blood-derived RNA-Seq data from both nonhuman primates and human cohorts. By contrasting EBOV-infected samples against a broad panel of viral and bacterial infections, including MPXV, influenza, bacterial pneumonia, HIV, and multiple SARS-CoV-2 variants, we addressed a central challenge in infectious disease transcriptomics: distinguishing pathogen-specific transcriptional signals from broadly shared host responses. Our results demonstrate that conventional differential expression analysis primarily captures generalized antiviral programs, whereas the integration of cross-dataset filtering, NanoString exclusion, cross-ranking, and downstream classification enables the identification of a transcriptional signature that exhibits specificity for EBOV across independent cohorts.

A key observation from the initial EBOV analysis was that many of the highest-ranking genes were classical interferon-stimulated genes that were also strongly activated across multiple infections. Genes such as *OAS1* and *IFI44L* ranked highly in the EBOV dataset but were consistently upregulated in MPXV, influenza, and SARS-CoV-2 cohorts. Although these genes are robust indicators of viral infection, their broad responsiveness limits their usefulness as EBOV-specific markers. This finding reinforces the importance of removing widely responsive immune genes when the goal is to derive a pathogen-specific signature rather than a general infection classifier.

By combining statistical selection using GLMQL-MAS with cross-infection filtering, NanoString panel exclusion, and a cross-ranking strategy, we derived a refined set of 281 EBOV-specific genes. From this pool, the top-50 EBOV-specific gene set demonstrated strong discriminatory performance in both the training and independent test cohorts. Principal component analysis and logistic regression models based on these genes achieved clear separation between EBOV and Non-EBOV samples, whereas models constructed using all genes showed substantial overlap. The marked improvement in F1 score during independent validation indicates that restricting the feature space to biologically prioritized genes enhances generalizability. Furthermore, the use of a simple linear classifier combined with dimensionality reduction to two principal components constrains model complexity and reduces the likelihood of overfitting, which is a persistent concern in high-dimensional transcriptomic analyses.

Functional enrichment analysis provides additional biological insight into the EBOV-specific signature. In contrast to broadly shared antiviral pathways dominated by interferon signaling, the top-50 EBOV-specific genes were enriched for vascular, secretory, and coagulation-related processes, including platelet activation, fibrinolysis, and leukocyte transendothelial migration. Metabolic pathways were also represented. This enrichment pattern suggests that EBOV infection may induce coordinated alterations in vascular function, extracellular remodeling, and host metabolism, which extend beyond canonical antiviral defense mechanisms.

Among the EBOV-specific genes, *ADAMTS1* emerged as a particularly distinctive candidate. In our analysis, *ADAMTS1* was consistently upregulated in EBOV-infected samples across both training and independent test datasets, whereas it remained neutral or downregulated in comparator infections. This directional specificity distinguishes *ADAMTS1* from classical interferon-driven genes. Although its precise mechanistic role requires further investigation, the consistent and infection-specific regulation of *ADAMTS1* supports its potential value as a discriminatory biomarker and highlights biological processes that may be uniquely amplified during EBOV infection.

An important methodological feature of this study is the decision to perform gene selection independently within each dataset prior to any cross-cohort integration. This design reduces bias that could arise from batch correction during differential expression analysis and preserves within-study biological structure. Batch correction was applied only after candidate genes were identified, which enables cross-cohort visualization and classification without influencing primary statistical testing.

In summary, this study introduces a structured filtering and ranking pipeline that identifies EBOV-specific transcriptional signatures from heterogeneous blood RNA-Seq datasets. By prioritizing genes that remain non-responsive across multiple comparator infections while retaining strong EBOV-associated differential expression, the approach reveals host pathways linked to vascular regulation, secretion, and metabolic processes rather than generalized antiviral signaling. These findings underscore the importance of cross-pathogen comparison in defining disease-specific biomarkers and provide a framework that can be extended to other emerging infectious diseases.

### 4.1 Limitations and future directions

Several limitations of this study should be acknowledged. First, although both nonhuman primate and human-derived datasets were included, the identification and optimization of the EBOV-specific gene set relied primarily on nonhuman primate infection models. While these models closely recapitulate many aspects of Ebola virus disease in humans, species-specific differences in immune regulation may influence transcriptional responses. As such, the EBOV-associated gene set identified here should be viewed as hypothesis-generating rather than definitive.

Second, the number of available human EBOV transcriptomic datasets remains limited, particularly those with well-matched controls, longitudinal sampling, and standardized sequencing protocols. This constraint necessitated the integration of datasets generated across different platforms, study designs, and populations, which may introduce residual technical or biological variability despite careful normalization and filtering. Although the strong concordance observed between training and independent test datasets supports the robustness of the findings, broader validation across additional human cohorts will be required to confirm generalizability.

An additional consideration is that publicly available cohorts were derived from both whole-blood and PBMC RNA-seq preparations, which differ in cellular composition and may influence transcriptional profiles. Future studies using harmonized sampling strategies across pathogens, ideally relying on consistently generated whole-blood or PBMC datasets, may further refine identification of EBOV-specific transcriptional signatures and improve cross-cohort comparability.

Importantly, this study does not establish causal roles for the identified genes in EBOV pathogenesis. The observed transcriptional patterns reflect associations with infection status rather than mechanistic function. Functional validation using targeted experimental systems, such as *ex-vivo* human immune cells or organoid-based models, will be necessary to determine whether these genes actively contribute to disease processes or represent downstream effects of infection.

Future work should therefore focus on validating and refining the proposed EBOV-specific gene set using expanded, well-controlled human-derived datasets, ideally including diverse populations, early infection time points, and clinically relevant comparators. Integration with proteomic, metabolomic, and clinical outcome data may further enhance biological interpretation and translational relevance.

## 5 Conclusion

In this study, we developed a cross-dataset filtering and ranking framework to identify EBOV-specific transcriptional signatures from heterogeneous blood-derived RNA-Seq datasets. By systematically excluding genes that exhibit broad activation across multiple viral and bacterial infections, our approach moves beyond conventional differential expression analysis, which often captures generalized antiviral responses rather than pathogen-specific biology. The resulting EBOV-specific gene set demonstrated strong and reproducible discriminatory performance across independent cohorts, which supports the robustness and generalizability of the proposed pipeline.

Functional enrichment analysis revealed that the EBOV-specific signature is associated primarily with vascular, coagulation-related, and secretory pathways, rather than canonical interferon-dominated antiviral signaling. These findings suggest that EBOV infection induces coordinated alterations in host vascular regulation and extracellular remodeling, which may represent defining features of EBOV-associated host responses. Within this signature, *ADAMTS1* emerged as a distinctive gene showing consistent upregulation in EBOV while remaining largely downregulated or transcriptionally inactive across comparator pathogens, which highlights its potential value as an EBOV-specific biomarker candidate.

Beyond the specific case of EBOV, the framework presented here provides a generalizable strategy for deriving pathogen-specific host-response signatures through cross-pathogen comparison. This approach may support the development of more precise host-based diagnostic tools capable of distinguishing infections with overlapping clinical and transcriptional profiles. Future studies integrating longitudinal clinical samples and functional validation will be important to clarify the mechanistic contributions of identified genes and to evaluate their translational potential in real-world diagnostic settings.

## Data Availability

All RNA-Seq datasets used in this study are publicly available through the NCBI Gene Expression Omnibus (GEO). All datasets can be accessed at https://www.ncbi.nlm.nih.gov/geo/. The EBOV training dataset is available under accession GSE103825. The independent EBOV test dataset is available under accession GSE115785. Non-EBOV comparator and filtering datasets include MPXV (GSE234118), influenza and bacterial pneumonia (GSE161731), severe COVID-19 (original strain; GSE171110), SARS-CoV-2 Beta variant PBMC dataset (GSE189039), SARS-CoV-2 Omicron (GSE201530), and acute HIV-1 infection (GSE199911). Independent non-EBOV test cohorts include seasonal influenza datasets (GSE158592, GSE155635, GSE196350, GSE213168), a multinational bacterial and influenza host-response cohort (GSE211567), and an additional clinical transcriptomic dataset comparing SARS-CoV-2 and influenza infections (PMC8202013). All data were generated by the original study authors and are used here in accordance with their public availability.
